# Transtibial pullout repair improved short-term clinical outcomes in patients with oblique medial meniscus posterior root tear comparable to radial root tear

**DOI:** 10.1007/s00590-024-04137-9

**Published:** 2024-11-28

**Authors:** Naohiro Higashihara, Takayuki Furumatsu, Yuki Okazaki, Yusuke Yokoyama, Masanori Tamura, Koki Kawada, Tsubasa Hasegawa, Toshiki Kohara, Toshifumi Ozaki

**Affiliations:** 1 Department of Orthopaedic Surgery, Okayama University Graduate School of Medicine, Dentistry, and Pharmaceutical Sciences, 2-5-1 Shikatacho, Kitaku, Okayama, 700-8558 Japan; 2 Department of Orthopaedic Surgery, Okayama Red Cross Hospital, 2-1-1 Aoe, Kita-Ku, Okayama City, Okayama, 700-8607 Japan

**Keywords:** Clinical outcomes, Medial meniscus, Oblique tear, Posterior root tear, Pullout repair, Radial tear

## Abstract

**Purpose:**

Medial meniscus (MM) posterior root tears (PRT) can lead to excessive knee loading and unsatisfactory clinical outcomes after non-operative treatment or meniscectomy. Although favourable clinical outcomes after MM posterior root (PR) repair have been reported, no study has specifically investigated the outcomes of different types of MMPRT. This study aimed to compare the clinical outcomes of patients with complete radial and oblique MMPRT following MMPR repair.

**Methods:**

Forty patients who had undergone MMPR repair were retrospectively investigated. Patients with type 2 (20 knees) and 4 MMPRT (20 knees) were included in this study. The MMPRT type was classified according to the LaPrade classification. Plain radiographs, magnetic resonance images, arthroscopic findings, and pre- and postoperative clinical outcomes were evaluated.

**Results:**

At 1 year postoperatively, clinical outcomes notably improved in patients with type 2 and 4 MMPRT. No significant differences were observed in any of the evaluations between these patients, both before and after the surgery.

**Conclusion:**

Patients with type 2 and type 4 MMPRT exhibited significantly improved clinical outcomes. MMPR repair is beneficial in treating type 2 and type 4 MMPRT.

**Level of evidence:**

IV

## Background

The medial meniscus (MM) posterior root (PR) anchors the meniscus to the bone, playing a crucial role in preventing excessive loading during knee motion and weight-bearing [1]. MM posterior root tears (PRT) are a meniscal injury that develops due to minor trauma against the background of meniscal degeneration [2]. MMPRT interrupt the continuity of circumferential fibres [3], leading to the early progression of knee osteoarthritis (OA) due to progressive medial meniscus extrusion (MME), excessive loading, and loss of meniscal function [4–7]. Given that joint degeneration progresses and symptom relief are limited after conservative treatment [8, 9], early diagnosis and surgical repair are effective to halt the rapid progression of knee OA and attain favourable postoperative outcomes [10, 11].

**Fig. 1:**
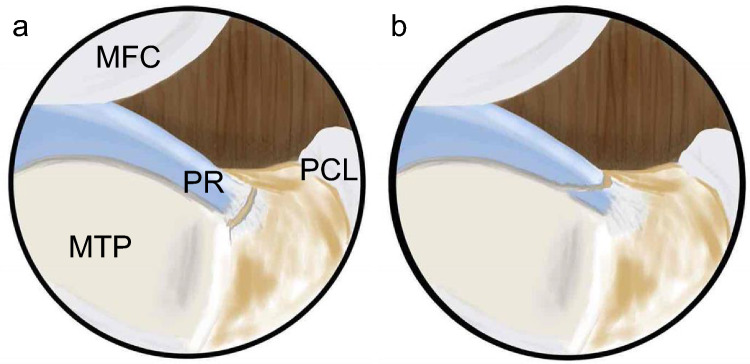
Schematic diagram of tear types according to the LaPrade classification [10]. **a** Type 2: complete radial tear of the posterior root. **b** Type 4: complete oblique tear of the posterior root. MFC, medial femoral condyle; MTP, medial tibial plateau; PCL, posterior cruciate ligament; PR, posterior root

MMPRT can be classified into five types based on tear morphology, as reported by LaPrade et al. [12]. The most frequent types are complete radial tears (type 2) and complete oblique tears (type 4) based on the LaPrade classification [13]. A simple schema of the tear morphology is shown in Fig. 1. In type 4 MMPRT, a large remnant is left on the tibial side, and little ligamentous tissue remains on the main side of the meniscus. In such cases, healing between the MM posterior horn and the bone tunnel or between the MM posterior horn and the remnant may be anticipated. However, the MM posterior horn contains abundant chondromodulin-1 [14] and has disadvantages in terms of healing due to its limited blood supply. Thus, type 2 MMPRT may hold an advantage in terms of healing as ligamentous tissue remains on both the tibial and main meniscus sides.

MMPR repair reduces the mean tibiofemoral contact pressure by increasing the tibiofemoral contact area, as presented in a human cadaveric knee study [15, 16]. In clinical practice, transtibial pullout repair is commonly used because it has less technical difficulty and anatomical repair can be achieved [17]. However, to our knowledge, no study has yet delineated the outcomes of patients with various types of MMPRT.

The present study aimed to compare the clinical outcomes of patients with different types of MMPRT after pullout repair. We hypothesised that favourable clinical outcomes could be achieved for type 2 and 4 MMPRT after pullout repair and that clinical outcomes would be more favourable in patients with type 2 MMPRT than in those with type 4 MMPRT.

## Methods

### Study design

This study was approved by the Institutional Review Board of our university (Approval No.: 1537), and written informed consent was obtained from all patients.

A total of forty consecutive patients who underwent MMPR repair for MMPRT between October 2016 and December 2020 were retrospectively investigated. MMPRT was diagnosed based on characteristic magnetic resonance imaging (MRI) findings, including giraffe neck sign, cleft sign, medial extrusion sign, and ghost sign [18]. The inclusion criteria were as follows: femorotibial angle < 180°, Kellgren–Lawrence grades 0–2, no history of knee surgery, and high patient compliance. The exclusion criteria were as follows: type 1, 3, or 5 MMPRT; patient with arthritis such as reumatoid arthritis or other collagen disease; body mass index (BMI) > 30 kg/m^2^; and absence of either MRI or second-look arthroscopy findings. Finally, patients with type 2 MMPRT (20 knees) and type 4 MMPRT (20 knees) were included in this study (Table 1). Patient demographics, radiological measurements, second-look arthroscopic scores, and pre- and postoperative clinical outcomes were retrospectively evaluated. MMPRT types were classified according to the LaPrade classification [11].

**Table 1:** Patient demographics

	Type 2	Type 4	*P* value
Number of patients	20	20	
Age (years)	62.4 ± 9.9	65.1 ± 6.4	0.31^a^
Sex, male/female	9/11	4/16	0.18^b^
Height (m)	1.60 ± 0.1	1.57 ± 0.1	0.25^a^
Body weight (kg)	66.1 ± 10.6	63.1 ± 12.0	0.41^a^
BMI (kg/m^2^)	25.6 ± 3.0	26.0 ± 4.0	0.69^a^
Femorotibial angle (°)	176.8 ± 1.3	177.1 ± 1.9	0.71^a^
Medial tibial slope (°)	8.2 ± 2.8	8.4 ± 1.7	0.88^a^
Surgical technique MMA/TSS/TSS + PM suture	10/1/9	3/4/13	0.06^b^
Kellgren–Lawrence grade (0/1/2)	0/9/11	1/7/12	0.75^b^

Data on age, height, body weight, BMI, femorotibial angle, and medial tibial slope are presented as mean ± standard deviation

^a^Statistical differences in age, height, body weight, BMI, femorotibial angle, and medial tibial slope between the two groups were analysed using the *t*-test

^b^Statistical differences in sex, surgical technique, and Kellgren–Lawrence grade were analysed using Fisher’s exact test

*BMI* body mass index, *MMA* modified Mason–Allen suture, *TSS* two simple stitches, *PM* posteromedial

### Surgical procedure and second-look arthroscopic evaluation

Standard anteromedial and anterolateral portals were used for MMPR repair and second-look arthroscopy. Three different suturing methods were used. These methods included the modified Mason–Allen suture (MMA) with FAST-FIX (Smith and Nephew, Andover, MA, USA) (from 2016.12 to 2018.7), two simple stitches (TSS) with No. 2 Ultrabraid (Smith and Nephew) (from 2018.8 to 2018.11), and TSS and posteromedial suture (TSS + PM) with FAST-FIX (from 2018.11) [19–21]. Selection of the surgical procedure depended on when the surgery was performed. These three surgical techniques have been reported to yield positive clinical outcomes in 83 patients, with good arthroscopic healing scores documented [22]. A 4.5-mm tibial tunnel was created at the anatomical attachment of the MMPR using an MMPRT guide (Smith and Nephew). Tibial fixation of the pullout sutures was performed using a double-spike plate (Meira, Aichi, Japan) or Biosure RG interference screw (Smith and Nephew), with an initial tension of 20–30 N. All the surgeries were performed by a single experienced surgeon.

**Fig. 2:**
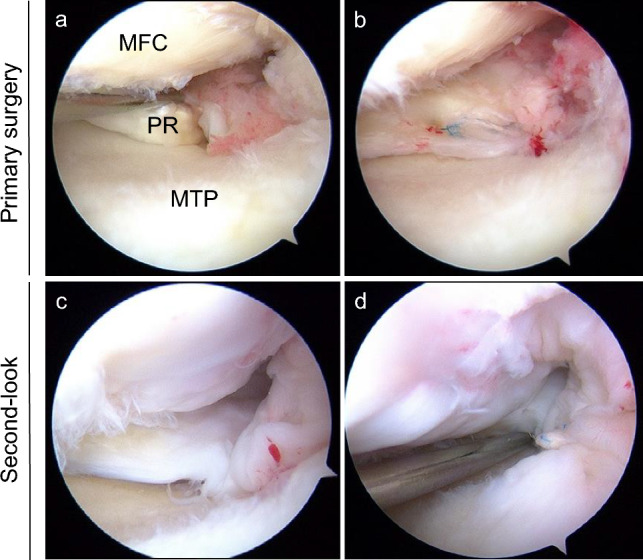
Intraoperative arthroscopic findings of type 2 medial meniscus (MM) posterior root (PR) tear in the left knee. **a** Confirmation of a type 2 MMPR tear. **b** MMPR repair using a modified Mason–Allen suture. **c** Complete healing of the MMPR scored at 10 out of 10 on second-look arthroscopic healing scores. Width (4, > 5 mm), stability (4, No lifting on probing at 20° of flexion), and synovial coverage (2, almost covered). **d** Stable MMPR showing no loosening at 20° flexion. MFC, medial femoral condyle; MTP, medial tibial plateau; PR, posterior root

**Fig. 3:**
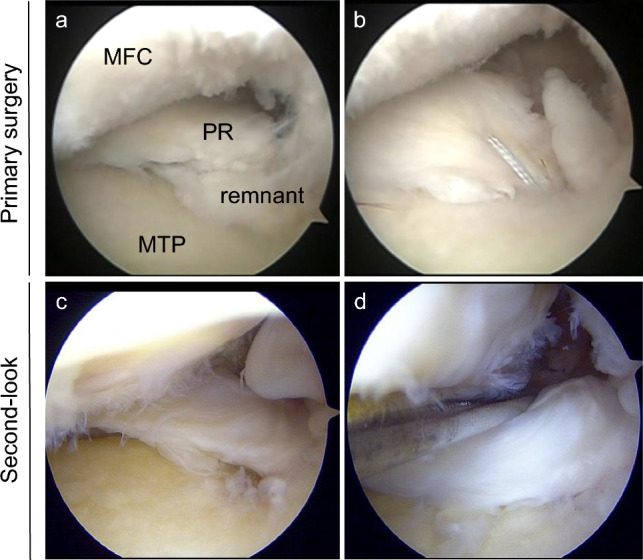
Intraoperative arthroscopic findings of type 4 medial meniscus (MM) posterior root (PR) tear in the left knee. **a** Confirmation of a type 4 MMPR tear. **b** MMPR repair using two simple stitches. **c** Complete healing of the MMPR scored at 10 out of 10 on second-look arthroscopic healing scores. Width (4, > 5 mm), stability (4, No lifting on probing at 20° of flexion), and synovial coverage (2, almost covered). **d** Stable MMPR showing no loosening at 20° flexion. MFC, medial femoral condyle; MTP, medial tibial plateau; PR, posterior root

Second-look arthroscopic assessment and removal of fixation devices were conducted 1 year postoperatively for all patients. The meniscal healing status was evaluated by a minimum of two orthopaedic surgeons during the surgery, utilising a semi-quantitative scoring system, ranging from 0 to 10 points [23]. The score involves three evaluation criteria: anteroposterior width (score: 0, 2, and 4), stability (score: 0–4), and synovial coverage of the repaired MMPR (score: 0–2). The most experienced surgeon in the operating team decided the final healing score with spot consultations (Figs. 2 and 3).

### Postoperative rehabilitation protocols

Following surgery, patients were instructed to maintain non-weight-bearing status, with their knees immobilised for a duration of 2 weeks. Subsequently, knee flexion exercises and partial weight-bearing were initiated gradually after this period. At 6 weeks post-surgery, full weight-bearing and knee flexion of up to 120° were permitted. Full flexion was allowed 3 months post-surgery.

### Clinical outcomes

The Lysholm score, International Knee Documentation Committee score, and Knee Injury and Osteoarthritis Outcome Score were evaluated immediately before primary surgery and second-look arthroscopy.

### MRI evaluations

MRI evaluations were performed before surgery and at 1 year postoperatively in the supine position, using Achieva 1.5 T (Philips, Amsterdam, the Netherlands), Excelart Vantage 1.5 T powered by Atlas (Canon Medical Systems corporation, Tochigi, Japan), or Oasis 1.2 T (Hitachi Medical, Chiba, Japan), with a coil under the 10° knee-flexed positions in a non-weight-bearing condition for all patients. The standard sequences of Oasis included a sagittal proton density-weighted sequence (repetition time/echo time: 1718/12) using a driven equilibrium pulse with a 90° flip angle and coronal T2-weighted multi-echo sequence (repetition time/echo time: 4600/84) with a 90° flip angle. The slice thickness was 4 mm with a 0-mm gap. The field of view was 16 cm, with an acquisition matrix size of 320 (phase) × 416 (frequency).

**Fig. 4:**
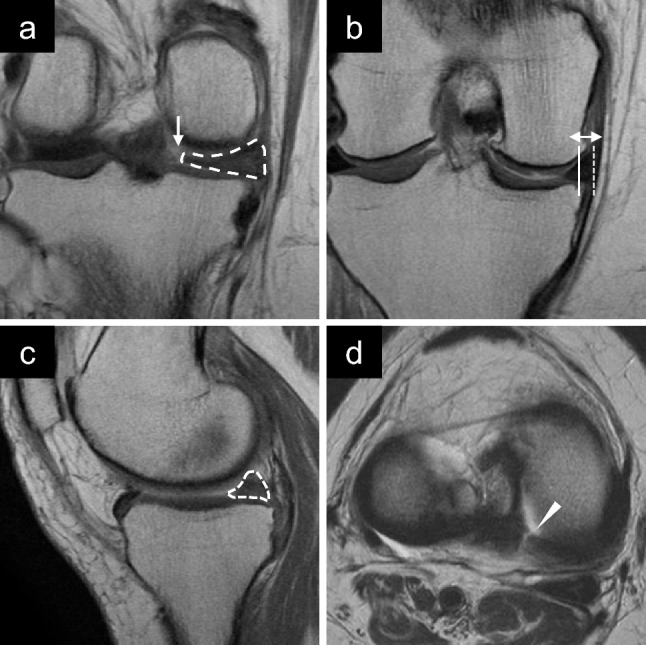
Preoperative magnetic resonance images of type 2 medial meniscus (MM) posterior root tears in the right knee. **a** Coronal view of the MM posterior portion showing a cleft sign (arrow) and a giraffe neck sign (dashed area). **b** Coronal view of the middle portion of the MM showing a medial extrusion (double arrow). **c** Sagittal view showing a ghost sign (dotted area). **d** Axial view shows a radial tear of the MM posterior root (arrowhead)

**Fig. 5:**
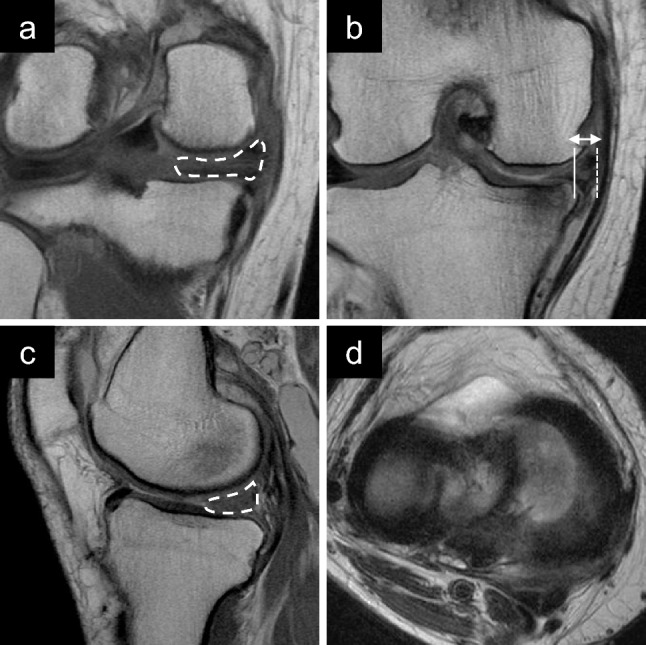
Preoperative magnetic resonance images of type 4 medial meniscus (MM) posterior root tears in the right knee. **a** Coronal view of the MM posterior portion showing a giraffe neck sign (dashed area). **b** Coronal view of the middle portion of the MM showing a medial extrusion (double arrow). **c** Sagittal view showing a ghost sign (dotted area). **d** Axial view showing no radial tears adjacent to the posterior root of the MM

On coronal images, MM body width was measured from the inner to the outer border of the MM that crossed the midpoint of the anteroposterior length of the MM. Absolute MME was measured from the medial margin of the tibial plateau, excluding osteophytes, to the outer border of the MM. Relative MME was calculated using the following formula: 100 × absolute MME/MM body width (Figs. 4 and 5).

### Statistical analysis

Data are presented as the mean ± standard deviation. Statistical differences between types 2 and 4 were analysed using the *t*-test, and differences between preoperative and postoperative data were examined using a paired *t*-test after confirming normal distribution using the Kolmogorov–Smirnov analysis. Differences in sex, surgical technique, and Kellgren–Lawrence grades were analysed using Fisher’s exact test. All statistical analyses were performed using EZR (Saitama Medical Center, Saitama, Japan), a graphical user interface for R (The R Foundation for Statistical Computing) [24], with the significance level set at *P* < 0.05.

## Results

Patient demographics and clinical characteristics are summarised in Table 1. Forty patients (13 men, 27 women) were included in this study. The mean age of the patients at the time of surgery was 63.7 years. The two groups showed no significant differences in all data, including age, BMI, femorotibial angle, and Kellgren–Lawrence grade, which are reported risk factors for poor postoperative clinical outcomes [25].

At 1 year postoperatively, significant improvements in clinical outcomes were observed in patients with both type 2 and 4 MMPRT (Tables 2 and 3) (*P* < 0.01* for all clinical scores). No significant difference was observed in clinical outcomes between types 2 and 4, either preoperatively or postoperatively (Table 4). Additionally, there were no significant differences in the MM body width, absolute MME, relative MME, or arthroscopic healing scores between types 2 and 4 (Table 5).

**Table 2:** Comparison of preoperative and postoperative clinical outcomes after pullout repair in patients with type 2 medial meniscus posterior root tears

		Preoperative	Postoperative	*P* value
KOOS	Pain	64.2 ± 17	87.0 ± 9.7	< 0.01*
	Symptoms	68.3 ± 15	83.5 ± 12	< 0.01*
	ADL	72.3 ± 16	89.5 ± 8.1	< 0.01*
	Sport/Rec	26.3 ± 22	56.2 ± 29	< 0.01*
	QOL	31.6 ± 17	64.8 ± 17	< 0.01*
Lysholm score		63.2 ± 14	87.8 ± 13	< 0.01*
IKDC score		41.4 ± 13	66.8 ± 13	< 0.01*

*KOOS* Knee Injury and Osteoarthritis Outcome Score, *IKDC* International Knee Documentation Committee, *ADL*, activities of daily living, *Sport/Rec* sports and recreation function, *QOL* knee-related quality of life

Data are expressed as mean ± standard deviation. Statistical differences between preoperative and postoperative data were analysed using a paired *t*-test. *P < 0.01

**Table 3:** Comparison of preoperative and postoperative clinical outcomes after pullout repair in patients with type 4 medial meniscus posterior root tears

		Preoperative	Postoperative	*P* value
KOOS	Pain	58.8 ± 22	86.2 ± 15	< 0.01*
	Symptoms	61.1 ± 21	83.2 ± 13	< 0.01*
	ADL	66.5 ± 21	88.0 ± 11	< 0.01*
	Sport/Rec	24.8 ± 23	58.0 ± 30	< 0.01*
	QOL	33.8 ± 24	71.8 ± 19	< 0.01*
Lysholm score		58.7 ± 13	86.4 ± 11	< 0.01*
IKDC score		35.4 ± 15	69.9 ± 17	< 0.01*

*KOOS* Knee Injury and Osteoarthritis Outcome Score, *IKDC* International Knee Documentation Committee, *ADL*, activities of daily living, *Sport/Rec* sports and recreation function, *QOL* knee-related quality of life

Data are expressed as mean ± standard deviation. Statistical differences between preoperative and postoperative data were analysed using a paired *t*-test. *P < 0.01

**Table 4:** Comparison of preoperative and postoperative clinical outcomes between medial meniscus posterior root tear types 2 and 4

			Type 2	Type 4	*P* value
Preoperative	KOOS	Pain	64.2 ± 17	58.8 ± 22	0.40
		Symptoms	68.3 ± 15	61.1 ± 21	0.23
		ADL	72.3 ± 16	66.5 ± 21	0.33
		Sport/Rec	26.3 ± 22	24.8 ± 23	0.83
		QOL	31.6 ± 17	33.8 ± 24	0.73
Lysholm score			63.2 ± 14	58.7 ± 13	0.31
IKDC score			41.4 ± 13	35.4 ± 15	0.20
Postoperative	KOOS	Pain	87.0 ± 9.7	86.2 ± 15	0.84
		Symptoms	83.5 ± 12	83.2 ± 13	0.95
		ADL	89.5 ± 8.1	88.0 ± 11	0.63
		Sport/Rec	56.2 ± 29	58.0 ± 30	0.85
		QOL	64.8 ± 17	71.8 ± 19	0.25
Lysholm score			87.8 ± 13	86.4 ± 11	0.71
IKDC score			66.8 ± 13	69.9 ± 17	0.53

*KOOS* Knee Injury and Osteoarthritis Outcome Score, *IKDC* International Knee Documentation Committee, *ADL*, activities of daily living, *Sport/Rec* sports and recreation function, *QOL* knee-related quality of life

Data are presented as mean ± standard deviation. Statistical differences between groups were analysed using the *t*-test

**Table 5:** Comparison of preoperative and postoperative magnetic resonance imaging measurements and arthroscopic meniscal healing scores on second-look arthroscopy between medial meniscus posterior root tear types 2 and 4

		Type 2	Type 4	*P* value
Preoperative	MMBW (mm)	8.8 ± 1.5	9.3 ± 2.1	0.43^a^
	aMME (mm)	3.4 ± 1.0	3.6 ± 1.5	0.59^a^
	rMME (%)	40.4 ± 14.2	39.7 ± 18.0	0.91^a^
Postoperative	MMBW (mm)	8.7 ± 1.7	8.7 ± 1.5	0.98^a^
	aMME (mm)	3.5 ± 1.2	3.7 ± 1.4	0.71^a^
	rMME (%)	42.1 ± 16.8	42.6 ± 13.7	0.85^a^
	ΔaMME (mm)	0.09 ± 0.8	0.1 ± 1.2	0.75^a^
Total arthroscopic healing score (3/4/5/6/7/8/9/10)		0/1/2/6/6/3/0/2	1/1/1/1/9/3/2/2	0.39^b^
AP width (0/2/4)		0/6/14	1/3/16	0.45^b^
Stability (0/1/2/3/4)		0/2/6/8/4	0/0/9/8/3	0.54^b^
Synovial coverage (0/1/2)		9/8/3	4/13/3	0.23^b^

*MMBW* medial meniscus body width, *aMME* absolute medial meniscus extrusion, *rMME* relative medial meniscus extrusion, *ΔaMME* postoperative aMME − preoperative aMME, *AP* anterior–posterior

Data on MMBW, aMME, rMME, and ΔaMME are presented as mean ± standard deviation

^a^Statistical differences in preoperative/postoperative MMBW, aMME, rMME, and ΔaMME between the groups were analysed using the *t*-test

^b^Statistical differences in arthroscopic healing scores were analysed using Fisher’s exact test

## Discussion

The primary finding of this study highlights that favourable clinical outcomes following pullout repair are attainable not only for type 2 MMPRT but also for type 4 MMPRT. In this study, the clinical outcomes significantly improved in the two groups; however, no significant differences were observed between them. Our hypothesis was partially confirmed.

Numerous types of meniscal tears occur, including radial, longitudinal, bucket-handle, and degenerative tears [26], with each tear pattern necessitating specialised treatment approaches. According to LaPrade et al., who classified MMPRT in 2015, a distinct distribution of tear morphologies also exists in MMPRT, suggesting that various treatment methods may be required for different types of MMPRT [12]. Several studies on MMPRT have described favourable clinical outcomes after pullout repair [11]; nevertheless, no study has focused on the clinical outcomes after pullout repair among different types of MMPRT. Clinical outcomes are similar between type 2 and 4 MMPRT; however, we hypothesised that type 4 MMPRT would have disadvantages in terms of healing because of the limited blood supply due to remnant characteristics and the greater strain on sutures caused by the extended distance between the remnant and tibial bone tunnel. The biological environment such as growth factors from the bone tunnel (bone marrow) [27] or a comparatively delayed rehabilitation protocol could potentially contribute to achieving favourable healing scores for both types of MMPRT at 1 year postoperatively.

In the present study, three different surgical techniques were used: MMA, TSS, and TSS + PM. We found that the clinical outcomes were consistent with those reported in a previous study [28]. In this previous study, patients treated using these three surgical techniques showed similar clinical outcomes, including arthroscopic healing scores. The number of each type (type 2 and 4 MMPRT) included was 24 and 3 for MMA, 23 and 4 for TSS, and 21 and 2 for TSS + PM, respectively. We contend that the type of surgical techniques utilised in this study had minimal impact on the clinical outcomes. The three pullout techniques demonstrated effectiveness for both type 2 and type 4 MMPRT. Consequently, standard pullout repair without supplementary sutures or devices remains viable, even when type 4 MMPRT is suspected based on preoperative MRI findings.

Although a low initial weight-bearing line ratio, high BMI, severe OA, and large MME have been identified as risk factors for poor clinical outcomes following MMPRT [25, 29, 30], the findings of this study suggest that MMPR pullout repair proves to be an effective surgical treatment for both type 2 and 4 MMPRT. Despite the great clinical outcomes after the surgery [31], some unfavourable data exist regarding the ability of this technique to reduce MME on postoperative MRI [32, 33]. However, previous research has shown that pullout repair can yield satisfactory clinical results even if MME does not improve postoperatively [34, 35]. Similarly, MME did not improve postoperatively in our study; however, the postoperative clinical outcomes significantly improved in both groups. Based on the results of the present study, type 4 MMPRT does not emerge as a risk factor for poor clinical outcomes. Additionally, good healing with respect to arthroscopic stability was noted in both groups. We believe that type 4 MMPRT is unlikely to lead to more rapid progression to severe OA compared to type 2 MMPRT; nevertheless, mid- to long-term follow-up is warranted.

This study had some limitations. First, it had a relatively small sample size and was not blinded. We cannot completely rule out the possibility of a type II error in the finding that there was no difference in postoperative outcomes between type 2 and 4 MMPRT. For this reason, it is possible that inter-group differences were not detected; thus, further research is necessary. Second, the follow-up period was too short to demonstrate the long-term clinical outcomes. Additional studies involving long-term follow-up are required. Third, failure load of healed meniscal tissue was not measured in a biomechanical test. This will require animal experiments using the MMPRT model. Fourth, this study employed three different surgical techniques. However, as previously mentioned, we believe that the influence of these variations on the results is minimal.

## Conclusions

Significantly improved clinical outcomes were observed in patients with both type 2 and 4 MMPRT. Pullout repair is beneficial in treating both types of MMPRT. The findings of this study indicate that both type 2 and 4 MMPRT, which represent the majority of tear morphology, demonstrate reasonable outcomes with repair, probably regardless of the technique although our data are limited. This insight will facilitate the development of straightforward and comprehensible treatment strategies for MMPRT.

## Data Availability

The datasets used and/or analysed during the current study are available from the corresponding author on reasonable request.

## Abbreviations

BMI: Body mass index
MM: Medial meniscus
MMA: Modified Mason–Allen suture
MME: Medial meniscus extrusion
MRI: Magnetic resonance imaging
OA: Osteoarthritis
PR: Posterior root
PRT: Posterior root tears
TSS: Two simple stitches
